# Genomic Integrity Is Favourably Affected by High-Intensity Interval Training in an Animal Model of Early-Stage Chronic Kidney Disease

**DOI:** 10.1186/s40798-016-0055-y

**Published:** 2016-08-09

**Authors:** Patrick S. Tucker, Aaron T. Scanlan, Rebecca K. Vella, Vincent J. Dalbo

**Affiliations:** 1 Clinical Biochemistry Laboratory, School of Medical and Applied Sciences, Central Queensland University, Bruce Highway, Building 81, Room 1.11, Rockhampton, 4702 Queensland Australia; 2 Human Exercise and Training Laboratory, Central Queensland University, Bruce Highway, Building 81, Rockhampton, 4702 Australia; 3 Cardiovascular Pharmacology Laboratory, Central Queensland University, Bruce Highway, Building 81, Rockhampton, 4702 Australia

## Abstract

**Background:**

Chronic kidney disease (CKD) is an irreversible disease that diminishes length and quality of life. Emerging evidence suggests CKD progression and genomic integrity are inversely and causally related. To reduce health complications related to CKD progression, chronic aerobic exercise is often recommended. To date, appraisals of differing modes of exercise, along with postulations regarding the mechanisms responsible for observed effects, are lacking.

In order to examine the ability of aerobic exercise to encourage improvements in genomic integrity, we evaluated the effects of 8 weeks of high-intensity interval training (HIIT; 85 % VO_2max_), low intensity training (LIT; 45–50 % VO_2max_), and sedentary behaviour (SED), in an animal model of early-stage CKD.

**Methods:**

To assess genomic integrity, we examined kidney-specific messenger RNA (mRNA) expression of genes related to genomic repair and stability: *Fan1*, *Mre11a*, and telomere length as measured by T/S ratio.

**Results:**

Following HIIT, mRNA expression of *Fan1* was significantly up-regulated, compared to SED (*p* = 0.026) and T/S ratio was significantly increased, compared to SED (*p* < 0.001) and LIT (*p* = 0.002).

**Conclusions:**

Our results suggest that HIIT is superior to SED and LIT as HIIT beneficially influenced the expression of genes related to genomic integrity.

## Key Points

1. CKD progression and genomic integrity are inversely and causally related.
2. To reduce complications related to CKD progression, chronic aerobic exercise is often recommended. Recently, clinicians and researchers have suggested that high-intensity interval training may be more beneficial, relative to low intensity training.
3. Following high-intensity interval training, mRNA expression of *Fan1* was significantly up-regulated, relative to sedentary behaviour (*p* = 0.026) and T/S ratio was significantly increased, relative to sedentary behaviour (*p* < 0.001) and low intensity training (*p* = 0.002).
4. Our results suggest that high-intensity interval training is superior to sedentary behaviour and low-intensity training; high-intensity interval training beneficially influenced the expression of genes related to the preservation of genomic integrity.

## Background

Chronic kidney disease (CKD) is an irreversible disease, characterised by a progressive decline in kidney function. Patients with end-stage renal disease (stage 5 CKD) are required to undergo renal replacement therapy (RRT), a term referring to life-long dialysis or kidney transplant. RRT represents a substantial financial burden on the Australian healthcare system, with yearly per-patient expenditure averaging nearly $55,000 (AUD) in 2012 [1].

While increases to RRT-related expenditure are troubling, the physiological consequences of CKD are most worrisome. The risks of comorbidity and mortality increase exponentially as renal function declines, resulting in a life expectancy that is decreased to such an extent that many CKD patients do not survive long enough to require RRT [2, 3].

The primary mechanisms responsible for the progression of CKD are revealed in the interactions between free radicals and reactive oxygen species (ROS), renal DNA, and inflammation mediators. For example, renal ROS, such as hydroxyl radicals, harmfully interact with nuclear and mitochondrial DNA bases [4–6]. This ROS-mediated damage to renal DNA may result in (a) inter-strand DNA, intra-strand DNA, and protein-DNA crosslinking that blocks DNA replication, leading to cell death [5–7] or (b) DNA strand breakage which results in cell death, if unrepaired, or genomic deletions and mutations, if repaired incorrectly [5–7]. ROS-mediated damage also activates the inflammatory response, in an effort to repair cellular and molecular injuries, which may encourage downstream DNA damage via the release of superoxide that is born of phagocytic activity [7–9]. Given the cyclical nature of this process, it becomes apparent that reducing and repairing DNA damage in the kidney is essential in terms of slowing CKD progression (Fig. 1).

**Fig. 1:**
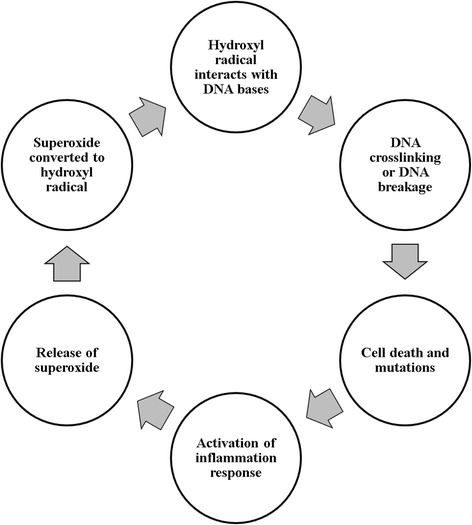
The primary mechanisms responsible for the progression of CKD: interactions between free radicals and reactive oxygen species, renal DNA, and inflammation mediators

Fortunately, mechanisms exist to repair damaged DNA. The Fanconi Anaemia (FA) pathway is a repair pathway devoted to repairing DNA inter-strand crosslinks (ICL) [10]. A crucial component of the FA pathway, FA-associated nuclease 1 (FAN1), a protein encoded by a gene of the same name (*Fan1*), makes nucleolytic incisions around the ICL [11, 12], thereby allowing additional molecules to complete repairs to damaged DNA. Evidence suggests depletion or mutation of FAN1 protein increases ICL [13], apoptosis [14], and kidney damage [14]. Unfortunately, ICL are not the only manifestation of DNA damage; DNA double-strand breaks are another form of damage that may result in genomic corruption.

Meiotic recombination 11 homolog A (MRE11A) is a nuclear protein involved in the repair of DNA double-strand breaks [15]. Encoded by the gene *Mre11a*, this protein is the core of the Mre11-Rad50-Nbs1 (MRN) complex, providing the nuclease activity that is required for nonhomologous joining of DNA ends [15]. Evidence suggests the MRN complex is activated by hypertonic, hyperosmotic salt in renal tissue [16], and cisplatin-induced nephrotoxicity [17], allowing for the repair of double-strand breaks in renal tissue that would otherwise suffer DNA damage.

*Mre11a* gene expression also plays a role in telomere preservation, via MRN-mediated ataxia-telangiectasia mutated kinase signalling in response to telomere shortening or telomere damage [18, 19]. The rate at which telomeres become depleted is not constant, allowing telomere length to serve as a predictor of life expectancy [20] and mortality [21]. Evidence suggests that patients with CKD have shorter telomeres and accelerated telomere shortening, relative to healthy counterparts [22, 23].

Despite their influence on molecular perturbations associated with CKD progression, few studies have examined the messenger RNA (mRNA) expression of *Fan1*, *Mre11a*, and telomere length in the context of CKD [14, 17, 22]. Furthermore, data describing potential modulators of mRNA expression of *Fan1*, *Mre11a*, and telomere length, in the context of CKD progression, are unavailable. Chronic aerobic exercise training has emerged as a promising therapy in terms of reducing CKD-related damage and slowing CKD progression [24–27]. Recently, clinicians and researchers have suggested that high-intensity interval training (HIIT) (generally, ≈85 % VO_2max_) may elicit greater benefits, relative to traditional light intensity training (generally, ≈50 % VO_2max_) [27–29]. However, the underlying mechanisms that allow for this have yet to be fully described. The purpose of the present study was to determine the effect of chronic HIIT (four times per week for 8 weeks) on the kidney-specific mRNA expression of molecules associated with genomic integrity (*Fan1*, *Mre11a*, and telomere length as measured by T/S ratio) in an animal model of early-stage CKD. It was hypothesised that HIIT would exert a more beneficial effect on *Fan1*, *Mre11a*, and T/S ratio, relative to sedentary behaviour (SED) and low intensity training (LIT).

### Note

In the discussion of this manuscript, data pertaining to genomic integrity are considered alongside previously reported data pertaining to CKD-related clinical markers (plasma albumin and kidney weight-body weight ratios) [30] and renal-specific mRNA expression of antioxidant enzyme genes (superoxide dismutase and catalase) [31] in the same animals.

## Methods

### Animals and General Overview

Male spontaneously hypertensive rats (SHR) (*n* = 39) (Animal Resource Centre, Canning Vale, WA, Australia) were housed in a temperature-controlled room (22–25 °C) with a dark-light cycle of 12:12 h and provided ad libitum access to standard laboratory chow and water. At the time of arrival, rats were 4–5 weeks old. Following a 2-week habituation period, allowing for acclimatisation to their new environment, all rats underwent a unilateral nephrectomy (6–7 weeks old). Following surgery, rats were allowed to recover for 2 weeks prior to the initiation of an 8-week exercise intervention. Blood pressures, body weights, and body lengths were recorded at pre-surgery, baseline (pre-exercise), 4 weeks (mid-intervention), and 8 weeks (post-intervention). Following the 8-week intervention, rats were euthanised 24 h following their last bout of exercise and tissues were collected for subsequent analysis. All research procedures were granted prior approval by an institutional Animal Ethics Committee (Animal Ethics Committee of Central Queensland University, project A13/11-305). This study was supported using internal funds awarded as part of Research Higher Degree study at Central Queensland University.

### Anesthetisation and Euthanasia Procedures

Prior to nephrectomy, rats were anesthetised with tiletamine/zolazepam (Zoletil) (25 mg/kg) and xylazine (10 mg/kg). For pain relief, meloxicam (2 mg/kg) was injected once daily for 2 days following nephrectomy. Prior to blood pressure readings, rats were anesthetised with tiletamine/zolazepam (25 mg/kg). Saline was injected subcutaneously following each anesthetisation to help avoid dehydration. Prior to being euthanised, rats were given a pentobarbitone sodium (Lethabarb) injection (1.5 mg/kg).

### Exercise Protocol

Rats were randomly assigned to one of three treatment groups: sedentary (SED, *n* = 12), low-intensity training (LIT, *n* = 13), and high-intensity interval exercise (HIIT, *n* = 14). The number of animals assigned to each group was dependant on the form and intensity of exercise, as the possibility existed that aerobic exercise may have proven too strenuous, resulting in the need to withdraw an animal from the experiment. However, there were no adverse events in any group, suggesting that aerobic exercise was well-tolerated in this disease model.

At 8–9 weeks of age, rats in the LIT and HIIT groups began treadmill running for 8 weeks. An identical 5-min warm-up (15 m/min) was performed by both training groups before each exercise session. Rats in the LIT group exercised at a treadmill speed of 15 m/min with an incline of 1° (45–50 % VO_2max_) [32], up to 33 min per day, 4 days per week, for 8 weeks. Rats in the HIIT group exercised at a treadmill speed of up to 50 m/min with an incline of up to 10° (≈85 % VO_2max_) [32], 30 min per day (2-min stationary period followed by 1-min sprint × 10 repetitions), 4 days per week, for 8 weeks. To permit adequate adaptation to treadmill running, thereby allowing each exercise session to be completed in its entirety, exercise training was progressive. By the beginning of the fourth week of training, all rats were performing exercise sessions at maximum intensity/duration—the exercise effort remained constant for the remaining 5 weeks of training. In an effort to standardise variables related to exercise training, while simultaneously allowing for the comparison of different intensities, exercise sessions for LIT and HIIT groups were matched based on distance travelled per session. That is, despite the difference in exercise intensity between groups, LIT and HIIT rats travelled the same distance (±1 m) during each exercise session, with distances progressing in unison from ≈200 m per session in week 1 to ≈500 m per session in week 8 (Table 1—republished with permission [27]).

**Table 1:** Detailed description of 8-week exercise protocol. Reproduced from Tucker et al. [27] with permission

Group	Variable	Measure	Wk1	Wk2	Wk3	Wk4	Wk5	Wk6	Wk7	Wk8
SED	NA	NA	NA	NA	NA	NA	NA	NA	NA	NA
LIT 45–50 % VO_2max_	Max. speed	Meters/min	15	15	15	15	15	15	15	15
	Incline	Degrees	1	1	1	1	1	1	1	1
	Duration	Minutes	13.3	20	26.6	33.3	33.3	33.3	33.3	33.3
	Intensity	Constant treadmill speed and constant treadmill incline								
	Frequency	Bouts/week	4	4	4	4	4	4	4	4
	Distance per bout	Meters	199.5	300	399	499.5	499.5	499.5	499.5	499.5
	Distance per week	Meters	798	1200	1596	1998	1998	1998	1998	1998
ᅟ										
HIIT ≈85 % VO_2max_	Max. speed	Meters/min	20	30	40	50	50	50	50	50
	Incline	Degrees	5	10	10	10	10	10	10	10
	Duration	minutes	30	30	30	30	30	30	30	30
	Intensity	2-min stationary period followed by 1-min sprint period × 10 reps								
	Frequency	Bouts/week	4	4	4	4	4	4	4	4
	Distance per bout	Meters	200	300	400	500	500	500	500	500
	Distance per week	Meters	800	1200	1600	2000	2000	2000	2000	2000

Exercise groups performed identical 5 min warm-up (15 m/min) prior to each exercise bout

*SED* sedentary, *LIT* light-intensity exercise, *HIIT* high-intensity interval training, *Wk* week

### Tissue Homogenisation and mRNA Isolation

Tissue used for analyses were chosen based on tissue-specific transcriptomic work performed in SHR [33]. In this study, renal tissue consisted specifically of renal cortex as mRNA is differentially expressed dependant on the type of renal tissue [34]. Approximately 100 mg (±10 mg) of tissue from the renal cortex was homogenised in 1 ml of TRI-reagent, using the TRI-reagent method (Sigma Aldrich, St. Louis, MO, USA). Samples were then centrifuged at 12,000×*g* for 10 min at 4 °C. The resulting supernatant was transferred to a new microtube and incubated for 5 min, at room temperature, to allow for disassociation of the nucleoprotein complex. Following incubation, 0.2 ml of chloroform was added before this solution was mixed and then incubated for 3 min. Samples were then centrifuged at 12,000×*g* for 15 min at 4 °C. The resulting supernatant was transferred to a new microtube. Following this separation, 500 μl of 2-propanol was added to each microtube. Microtubes were incubated at room temperature for 10 min and then centrifuged at 12,000×*g* for 10 min at 4 °C. The resulting supernatant was removed, leaving behind an mRNA pellet. The mRNA pellet was exposed to two ethanol washes (1 ml, 75 %). Following each wash, microtubes were vortexed and centrifuged at 7500×*g* for 5 min at 4 °C. The mRNA pellets were then allowed to air dry under a fume hood before being dissolved in 30 μl of RNase-free water. A 260/280 ratio was calculated to assess mRNA purity, with all ratios falling within commonly accepted parameters. The diluted mRNA samples were stored at −80 °C until later analyses.

### Total mRNA Determination and Reverse Transcription

Total mRNA concentrations from each sample were determined using a spectrophotometer (NanoDrop 2000c, Thermo Scientific, Wilmington, DE, USA). Next, mRNA concentrations were adjusted to 400 ng/μl by diluting the crude total mRNA extracts with RNase-free water. The standardised solutions of total cellular mRNA were reverse transcribed to synthesise complimentary DNA (cDNA) using a high-capacity cDNA reverse transcription kit (Applied Biosystems, Foster City, CA, USA). Briefly, ×2 RT master mix was prepared per kit instructions. Next, 10 μl of RT mix was added (and mixed via pipette trituration) to 10 μl of the standardised mRNA solution. These samples were placed on a thermal cycler (T100 Thermal Cycler, Bio-Rad, Gladesville, NSW, Australia) set to run at 25 °C for 10 min, 37 °C for 120 min, and 85 °C for 5 min. Following reverse transcription, the resulting cDNA template concentrations were diluted to 5 ng/μl by adding RNase-free water. The standardised cDNA solutions were frozen at −80 °C until quantitative reverse transcription polymerase chain reaction (qRT-PCR) was performed.

### Quantitative Reverse Transcription Polymerase Chain Reaction Analysis

Forward and reverse oligonucleotide primer pairs were developed using National Centre for Biotechnology Information’s (NCBI, Bethesda, MD, USA) Primer Designer Tool before being commercially synthesised (GeneWorks, Hindmarsh, SA, Australia) (Table 2). *Gapdh* was used as an internal reference gene for detecting relative change in the quantity of target mRNA due to its consideration as a constitutively expressed housekeeping gene following exercise training [35]. Following the examination of primer efficiencies, qRT-PCR reactions were mixed. Reactions contained the following mixture: 2.5 μl of prepared cDNA template, 5 μl of SYBR Select Master Mix (Life Technologies, Mulgrave, Victoria, Australia), 0.5 μl of sense and anti-sense primers, and 1.5-μl RNase-free water. Following the mixture of each reaction, qRT-PCR was performed with a thermal cycler (Rotor-Gene Q, Qiagen, Venlo, Netherlands). The amplification sequence involved an initial 10-min cycle at 95 °C, followed by 40 cycles, each composed of a denaturation step (95 °C for 15 s) and a primer annealing/extension step (60 °C for 45 s). Melting curves were performed to ensure sufficient PCR product was being generated by each primer, in the absence of primer dimers. All assays were performed in duplicate (coefficient of variation = 0.01).

**Table 2:** Primer sequences used in quantitative reverse transcription polymerase chain reactions

Target	Primer sequence (5′–3′)
Fan1 forward primer	CTTTATCAGCGAGCTGTGCG
Fan1 reverse primer	CACAGACTTGCCCATCCCAT
Mrella forward primer	CAAGGAAAACCCTGCACAGC
Mrella reverse primer	CCTAAGCCGTACAGAGCGAG
Telomere forward primer	GGTTTTTGAGGGTGAGGGTGAGGGTGAGGGTGAGGGT
Telomere reverse primer	TCCCGACTATCCCTATCCCTATCCCTATCCCTATCCCTA
Gapdh forward primer	GTTACCAGGGCTGCCTTCTC
Gapdh reverse primer	GATGGTGATGGGTTTCCCGT

*Fan1* Fanconi anaemia-associated nuclease 1, *Mre11a* meiotic recombination 11 homolog A, *Gapdh* glyceraldehyde 3-phosphate dehydrogenase

mRNA expression data were calculated using the Livak method [36], and data are presented as the fold change of the gene of interest, relative to that of control animals. Relative telomere length was assessed using methods described in detail elsewhere, [20, 37, 38]. Briefly, relative telomere length was quantified by determining the ratio (T/S ratio) of telomere repeat copy number (T) to single control gene copy number (S), relative to a reference sample. In this study, *Gapdh* was used as the single control gene.

### Clinical Measurements

Detailed methods describing the collection and analysis of clinical measurements, in the animals discussed in the current study, have been previously reported [27]. Briefly, clinical measures included systolic blood pressure, remnant kidney weight, remnant kidney weight to body weight ratio, and plasma levels of high-density lipoprotein, low-density lipoprotein, triglycerides, total cholesterol, albumin, and creatinine. These measurements receive consideration in the “Discussion” section of this manuscript as they help elucidate mechanisms that may relate to findings in the current study.

### Statistics

Data related to mRNA are presented as mean ± standard error of the mean (SEM). Data for mRNA expression were analysed using separate between-group one-way ANOVAs with Tukey’s post hoc comparisons. Analyses were performed using IBM SPSS Statistics (v20.0, IBM Corporation; Armonk, NY, USA). Statistical significance was set at *p* < 0.05.

## Results

### Clinical Measurements

Clinical measurements relating to the animals in the current investigation have been previously reported [27]. A summary of these results can be found in Table 3 (republished with permission).

**Table 3:** Previously reported clinical measures. Reproduced from Tucker et al. [27] with permission

Measure	Previously reported	Group	Mean	*p* value compare to HIIT	Mean duplicate CV
HDL (mg/ml)	Yes	SED	0.82 ± 0.13	0.109	0.06
		LIT	0.88 ± 0.15	0.591	
		HIIT	0.93 ± 0.12		
LDL (mg/ml)	Yes	SED	1.27 ± 0.27	0.285	0.03
		LIT	1.55 ± 0.34	0.001	
		HIIT	1.08 ± 0.32^a^		
TAG (mg/ml)	Yes	SED	5.65 ± 1.66	0.183	.04
		LIT	6.35 ± 2.65	0.029	
		HIIT	4.11 ± 2.04^a^		
CHOL (mg/ml)	Yes	SED	3.22 ± 0.47	0.263	NA
		LIT	3.71 ± 0.61	0.002	
		HIIT	2.83 ± 0.73^a^		
CRE (nmol/ul)	Yes	SED	1.07 ± 0.51	0.126	0.04
		LIT	2.06 ± 1.79	0.996	
		HIIT	2.11 ± 1.26		
ALB (mg/ml)	Yes	SED	26.23 ± 3.84	0.001	0.04
		LIT	29.88 ± 6.42	0.047	
		HIIT	35.24 ± 6.08^b^		
Kidney weight (g)	Yes	SED	1.57 ± 0.09	0.028	NA^c^
		LIT	1.59 ± 0.12	0.005	
		HIIT	1.46 ± 0.08^b^		
KW-BW ratio	Yes	SED	0.005 ± 0.0003	0.045	NA
		LIT	0.005 ± 0.0003	0.048	
		HIIT	0.0047 ± 0.0002^b^		
Post-In SBP (mmHg)	Yes	SED	239.77 ± 12.85	0.114	0.02
		LIT	241.63 ± 21.42	0.074	
		HIIT	227.12 ± 14.23		

Values presented as mean ± standard deviation

Values are re-reported with permission

Significance set at *p* < 0.05

*SED* sedentary, *LIT* light-intensity exercise, *HIIT* high-intensity interval training, *HDL* high-density lipoprotein, *LDL* low-density lipoprotein, *TAG* triglycerides, *CHOL* total cholesterol, *CRE* creatinine, *ALB* albumin, *KW-BW* kidney weight-body weight, *Post-Int SBP* post-intervention systolic blood pressure, *CV* coefficient of variation

^a^HIIT significantly different from LIT

^b^HIIT significantly different from LIT and SED

^c^CHOL is a calculated value and does not have a duplicate CV

### Quantitative Reverse Transcription Polymerase Chain Reaction Analysis

Results pertaining to renal expression of mRNA related to genomic health can be seen in Fig. 2a–c. In HIIT, mRNA expression of *Fan1* was significantly up-regulated, compared to SED (*p* = 0.026) and T/S ratio was significantly increased, compared to SED (*p* < 0.001) and LIT (*p* = 0.002). There were no significant between-group differences for *Mre11a* (HIIT v. SED, *p* = 0.184; HIIT v. LIT, *p* = 0.327).

**Fig. 2:**
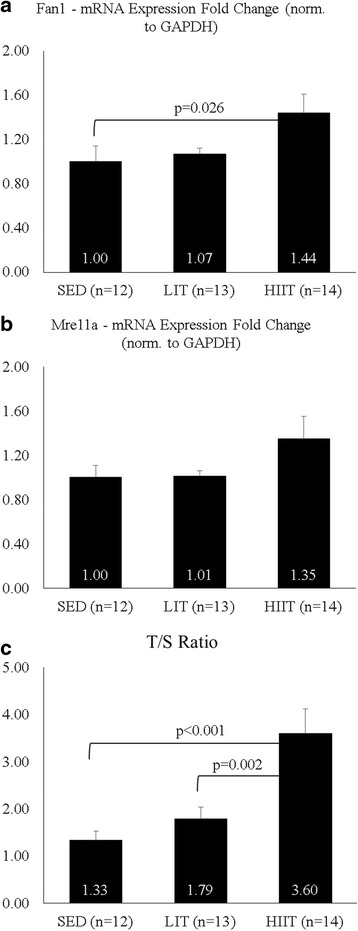
**a**–**c** mRNA related to genomic health—expression fold change

## Discussion

This is the first investigation to assess and compare the ability of SED, LIT, and HIIT to modulate kidney-specific mRNA expression of genes related to genomic integrity, in an experimental model of early-stage CKD. In fact, except for a handful of studies that have examined the relationship between chronic exercise and telomere length [39–43], we are unaware of any investigations that have assessed the ability of exercise, of any mode or duration, to modulate mRNA expression of genes related to genomic integrity (specifically, *Mre11a* and *Fan1*), in any tissue or disease model. As a result, little is known about the effects of chronic exercise on genes related to genomic integrity. There is, however, evidence to attest that chronic aerobic exercise can beneficially influence health-related markers and that these beneficial effects are further enhanced by increases in exercise intensity [29, 30, 44, 45]. This evidence provides a reasonable basis for two assumptions that are supported by the current findings: (a) some beneficial effects of chronic aerobic exercise may stem from genome-based genomic protection (i.e. genes that protect genes) and (b) some chronic exercise-mediated effects related to genomic protection may be further enhanced by increases in exercise intensity.

Using an animal model of progeroid aging that exhibits elevated mitochondrial DNA point mutations and systemic mitochondrial dysfunction, Safdar et al. reported 5 months of endurance exercise (15 m/min for 45 min, 3 times/week) mitigated mitochondrial DNA depletion and point mutations in the skeletal muscle, heart, and liver of mice [42]. However, the mechanisms that explain these positive, exercise-related effects remain unclear. Results from our study suggest that HIIT may preserve genomic integrity in the kidney, in part, by significant exercise-induced increases to renal expression of *Fan1* mRNA (v. SED). Unlike Safdar et al., our data suggests that changes to indices of genomic integrity may be intensity-dependant as HIIT was the only exercise condition that stimulated significant increases in *Fan1* mRNA expression (v. SED) and T/S Ratio (v. SED and LIT). However, there are several explanations that may account for the discrepancy between our data and data reported by Safdar et al., including training duration and disease model.

Work regarding *Fan1* gene expression has determined that the resultant protein is a required component of resistance against harmful DNA crosslinking [12–14] and is likely involved in other forms of DNA damage repair [46, 47]. This, combined with our current results, leads us to suggest that HIIT-induced increases in renal expression of *Fan1* mRNA are a beneficial adaptation that may partially explain other beneficial, renal-specific, HIIT-related effects in this group of animals. Specifically, we previously reported that plasma albumin and kidney weight-body weight ratio were significantly improved by HIIT (v. SED and LIT) in the same animals being examined in the current study [30]. This finding aligns with data that suggests blunted DNA repair, stemming from *Fan1* mutations or depletions, leads to interstitial nephritis [14], a condition associated with proteinuria and renal hypertrophy [48].

Renal T/S ratio, an indicator of telomere length, was also beneficially influenced by HIIT in the current study (v. SED and LIT). This may be partially due to non-significant increases in renal expression of *Mre11a* mRNA in HIIT. Research suggests that the MRN complex, whose core is encoded by the gene *Mre11a*, is required for the phosphorylation of TRF1, a telomeric repeat binding factor [19]. The phosphorylation of TRF1 results in the release of TRF1 from telomeres, thereby allowing telomerase, a ribonucleoprotein that adds the nucleotide “TTAGGG” to the 3′ end of telomeres, access to the ends of telomeres [19]. Simply put, the non-significant, HIIT-related increase in renal expression of *Mre11a* mRNA may have helped to preserve renal telomere length by allowing telomerase access to the ends of telomeres. That said, it is unlikely that non-significant increases in the renal expression of *Mre11a* mRNA fully explain the significant HIIT-related increases in renal T/S ratio (v. SED and LIT) seen in the current study.

Despite previous work performed in this area [39–41, 43], the mechanisms that allow for telomere preservation, following chronic aerobic exercise, are elusive. Similar to our results, previous work has suggested that chronic exercise can beneficially influence telomere length [40, 41]. What is less understood is the effect that exercise intensity has on telomere length. One study suggests that leukocyte telomere length is (1) preserved in adults who chronically and vigorously exercise and is (2) related to maximal aerobic capacity [40]. However, data from this study is cross-sectional and the exercise “intensity” discussed is not well defined [40]. Conversely, another study suggests that telomere length, in peripheral blood mononuclear cells, is best preserved by moderate levels of chronic physical activity, compared to low and high levels of physical activity [43]. However, this study categorized participants based on cross-sectional exercise energy expenditure data that was assessed via survey [43]. As such, and to our knowledge, this is the first study to explicitly examine the effect of exercise intensity on telomere length, regardless of tissue or disease model.

Aside from non-significant increases in the renal expression of *Mre11a* mRNA, there is evidence to suggest that significant HIIT-related increases in renal mRNA expression of antioxidant enzymes may help to explain the significant HIIT-related increase in renal T/S ratio. Reviewed elsewhere [49], telomeric DNA appears to be especially susceptible to attack by reactive oxygen species. We previously reported [31], in the same group of animals, that HIIT resulted in significant increases in the renal mRNA expression of the antioxidant enzymes superoxide dismutase (v. SED) and catalase (v. SED and LIT). Given the significant HIIT-related increases in the renal mRNA expression of superoxide dismutase (v. SED) and catalase (v. SED and LIT) [31], we suggest that significant HIIT-related increases to renal T/S ratio (v. SED and LIT) may have been influenced by concomitant increases in the renal mRNA expression of antioxidant enzymes. This assertion is supported by previous work performed in rats where increased oxidative stress was related to accelerated telomere shortening and decreased renal function [50].

## Conclusions

Our data indicated that HIIT was responsible for a significant up-regulation in the renal expression of *Fan1* mRNA (v. SED) and increases to renal T/S ratio (v. SED and LIT). The significant HIIT-related increases to renal T/S ratio may be partially explained by non-significant HIIT-related increases to renal expression of *Mre11a* mRNA. The significant HIIT-related increases to renal T/S ratio may also be partially explained by significant HIIT-related increases in renal mRNA expression of superoxide dismutase (v. SED) and catalase (v. SED and LIT). Combined with previously published data from our laboratory [30, 31], the current investigation provides compelling evidence that HIIT is superior to SED and LIT in regard to the preservation of genomic integrity in the kidney, making HIIT a promising therapy for patients with early-stage CKD. Clinical trials should be conducted in humans to further explore the use of HIIT as a means by which to slow disease progression in early-stage CKD populations.
